# Transmembrane TNF and Partially TNFR1 Regulate TNFR2 Expression and Control Inflammation in Mycobacterial-Induced Pleurisy

**DOI:** 10.3390/ijms19071959

**Published:** 2018-07-04

**Authors:** Husnu Uysal, Leslie Chavez-Galan, Dominique Vesin, Guillaume Blaser, Mahdia Benkhoucha, Bernhard Ryffel, Valérie F. J. Quesniaux, Irene Garcia

**Affiliations:** 1 Department of Pathology and Immunology, Centre Medical Universitaire (CMU), Faculty of Medicine, University of Geneva, 1211 Geneva, Switzerland; uysalhusnu@gmail.com (H.U.); lchavez_galan@iner.gob.mx (L.C.-G.); Marie-Dominique.vesin@unige.ch (D.V.); Guillaume.blaser@unige.ch (G.B.); Mahdia.Benkhoucha@unige.ch (M.B.); 2 Centre National de la Recherche Scientifique, UMR7355, and Experimental and Molecular Immunology and Neurogenetics, University of Orléans, 45100 Orléans, France; bryffel@cnrs-orleans.fr (B.R.); quesniaux@cnrs-orleans.fr (V.F.J.Q.)

**Keywords:** TNF, TNF receptors, BCG-induced pleurisy, inflammation

## Abstract

Pleural tuberculosis is one of the most frequent forms of extra-pulmonary tuberculosis observed in patients infected with *Mycobacterium tuberculosis.* Tumor Necrosis Factor (TNF) is a crucial cytokine needed to control tuberculosis infection that remains a leading cause of morbidity and mortality worldwide. TNF blockade compromises host immunity and may increase the risk of reactivation of latent infection resulting in overt pulmonary, pleural and extra-pulmonary tuberculosis. While TNF signaling is mainly considered pro-inflammatory, its requirement for the anti-inflammation process involved in the resolution of infection and tissue repair is less explored. Our study analyzes the role of TNF and TNF receptors in the control of the inflammatory process associated with *Bacillus Calmette-Guérin* (BCG)-induced pleurisy. This study shows that the absence of TNF causes exacerbated inflammation in the pleural cavity of BCG-infected mice which is controlled by the transmembrane TNF (tmTNF) expression. The lack of TNF is associated with an impaired cellular expression and shedding of TNFR2 in the pleural cavity. The presence of tmTNF restores the normal expression of TNFR2 on myeloid cells during BCG-induced pleurisy. We also show that absence of TNFR1 affects the expression of TNFR2 on pleural cells and inflammation in the pleural cavity of BCG-infected mice. In conclusion, tmTNF but not soluble TNF prevents pleural cavity inflammation leading to attenuation and the resolution of the inflammatory process caused by mycobacterial pleurisy in association with the expression of TNFR2 on myeloid cells.

## 1. Introduction

Tumor Necrosis Factor (TNF) is a pleiotropic cytokine exerting a large range of activities through its receptors, TNFR1 and TNFR2. TNF is first synthetized as a transmembrane molecule (tmTNF) and then cleaved by tumor necrosis factor-alpha converting enzyme (TACE) resulting in the soluble trimeric TNF molecule (solTNF). A majority of the TNF effector functions have been attributed to the solTNF form but many are also mediated by tmTNF, especially when cell-to-cell contact interaction is involved. Both solTNF and tmTNF as well as the Lymphotoxin-alpha (LT-α) signal through TNFR1 and TNFR2 [1,2]. Soluble TNFR1 and TNFR2 are also shed following proteolytic cleavage by TACE. Soluble TNF receptors (solTNFRs) are present in the blood of healthy individuals and during pathological situations such as infection and cancer; the dramatically increased levels of solTNFRs correlate with disease severity [3,4].

TNF is a master cytokine required for host responses against mycobacterial infections mainly caused by *Mycobacterial tuberculosis* (*M. tuberculosis*), which is still a major health problem [1,5,6,7]. TNF protective immunity against mycobacteria is mediated through TNFR1 while TNFR2 is mainly involved in tolerogenicity [8,9,10,11]. In particular, shed soluble TNFR2 neutralizes bioactive TNF, preventing signaling through TNFR1 [10]. Anti-TNF therapies have shown their efficacy for the treatment of autoimmune inflammatory diseases, however, an increased risk of reactivation of latent tuberculosis or new infections were observed in patients [12]. The pulmonary form is the most common form of tuberculosis infection in humans but extra-pulmonary accounts for about one third of reported tuberculosis cases [13]. Pleural tuberculosis can also be observed in cases of reactivation of a latent infection, and in certain cases, associated with the use of corticosteroid and anti-TNF treatments or presence of comorbidities as HIV/AIDS and diabetes [14]. 

*Mycobacterium bovis Bacillus Calmette-Guérin* (BCG) is the live attenuated mycobacterium used as a vaccine against *M. tuberculosis* infection. BCG vaccination promotes Th1 type immune responses and confers non-specific protection against non-related mycobacterial infections [15]. BCG is currently used for the immunotherapy of non-muscle-invasive bladder cancer [16]. We have previously used BCG infection for the study of the role of TNF in host defense mechanisms against attenuated mycobacteria [1]. A mouse model of BCG-induced pleurisy has allowed the evaluation of cell recruitment and specific functions of activated cells in the pleural cavity [17]. BCG-induced pleurisy was lethal in mice deprived of TNF and TNF receptors but mice expressing tmTNF survived to the BCG pleural infection [18]. The resistance of tmTNF expressing mice was mediated by myeloid-derived suppressor cells (MDSC) expressing tmTNF and interacting with T cells expressing TNFR2 but not TNFR1 [18].

TNF is considered one important pro-inflammatory cytokine due to its association with pathological processes. However, as many other molecules, a paradoxical role can be attributed indicating that TNF has to be synthesized at the good moment and intensity to trigger a TNF-dependent cascade leading to anti-inflammatory process and disease resolution [6]. The present study evaluates the contribution of TNF and TNFRs in the prevention and attenuation of the inflammatory process associated with BCG-induced pleurisy. Our data shows that tmTNF, TNFR2 and TNFR1 (indirectly) are critical for preventing inflammation during BCG-induced pleurisy.

## 2. Results

### 2.1. BCG-Induced Pleurisy Triggers the Accumulation of Inflammatory Cells Releasing Soluble TNFR2

**Figure 1:**
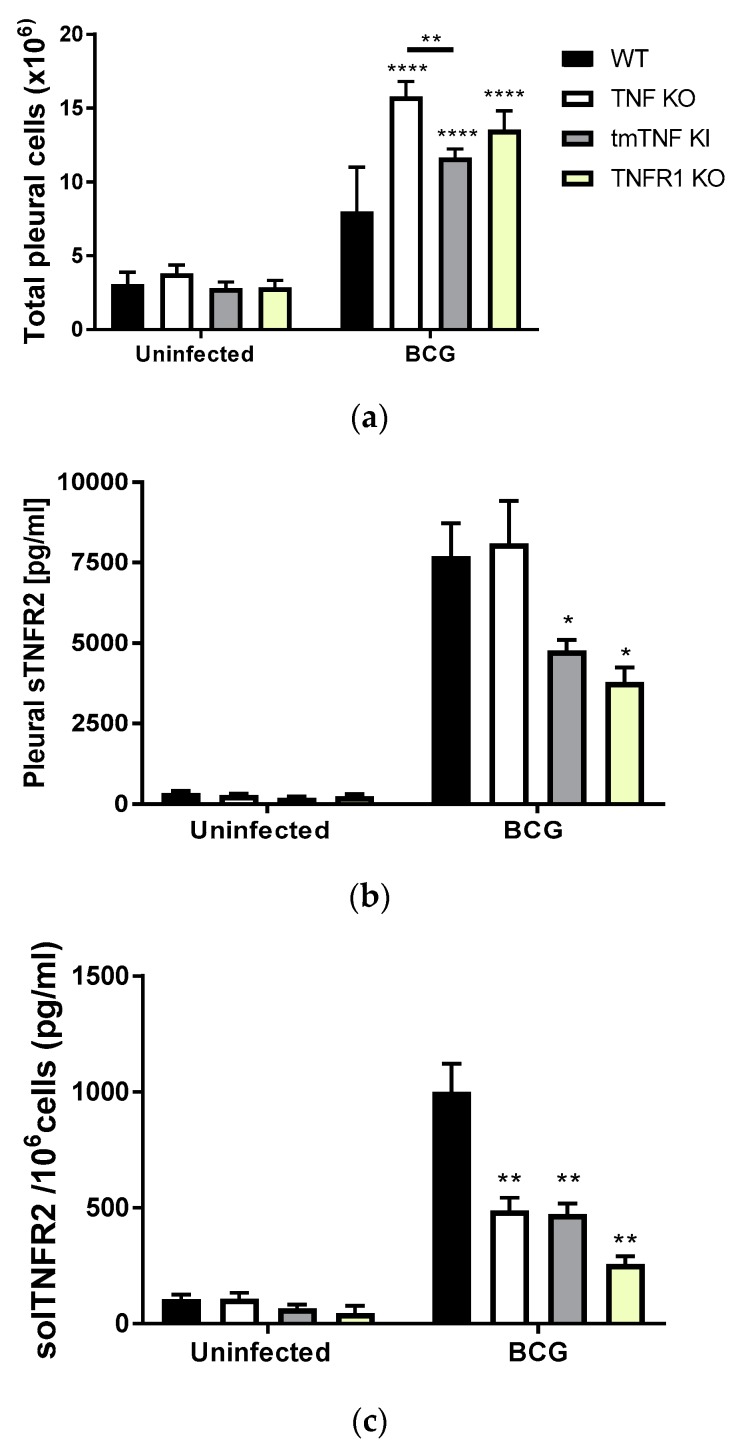
The BCG-induced pleurisy in mice causes the accumulation of pleural cells shedding soluble TNFR2 in the pleural fluid. (**a**) Total number of cells from the pleural cavity recovered from uninfected and from mice infected with BCG for 14 days. Data are represented as bar graphs of means ± SEM from four experiments (*n* = 6, uninfected mice and *n* = 12, infected mice/per group). Comparisons are done between infected WT mice versus infected mutant mice or as indicated in the figure. ** *p* < 0.01, and **** *p* < 0.0001 versus WT if not indicated otherwise; (**b**) Concentrations of soluble TNFR2 in the pleural cavity of uninfected mice or in mice infected inside the pleural cavity with BCG at day 14 post-infection. Data are represented as bar graphs of means ± SEM (*n* = 4 uninfected mice and *n* = 12 infected mice/group). Comparisons are between infected WT versus infected mutant mice, * *p* < 0.05; (**c**) Concentration of soluble TNFR2 reported by million cells from the pleural cavity of uninfected or BCG-infected mice as described in (b). Data are represented as bar graphs of means ± SEM. Comparisons are done between infected WT versus infected mutant mice or as indicated in the figure. ** *p* < 0.01.

We have reported that BCG-induced pleurisy is lethal in TNF KO and TNFR1TNFR2 KO mice [17]. In contrast, mice expressing only tmTNF (tmTNF KI) but not solTNF are able to resist the infection [18]. In the present study, we have first evaluated the cellular content in the pleural cavity of uninfected and BCG-infected mice at day 14 post-infection. WT, TNF KO, tmTNF KI and TNFR1 KO mice infected with one million living BCG in the pleural cavity showed an increased cell number compared to uninfected mice of the same genotype (Figure 1a). TNF KO, tmTNF KI as well as TNFR1 KO mice depicted a higher number of cells in the pleural cavity than WT mice. However, TNF KO exhibited the highest number, which was significantly higher than tmTNF KI mice (Figure 1a). We have already reported that TNFR1TNFR2 KO mice showed a similar number of myeloid and lymphoid cells than TNF KO mice in the pleural cavity following BCG-induced pleurisy [17]. 

Because BCG infection activates TNF pathways, membrane TNFRs are shed and the concentration of solTNFRs can be quantified in the serum as reported in Reference [19]. We asked whether BCG-induced pleurisy increased the level of solTNFR2 in the pleural fluid. We found higher concentrations of solTNFR2 in the pleural cavity of BCG-infected mice compared with uninfected mice, as expected. A significant increase of solTNFR2 was observed mainly in WT and TNF KO, and less so in tmTNF KI and TNFR1 KO, compared with the respective uninfected mice (Figure 1b). However, as mutant mice had higher cell numbers in the pleural cavity, we calculated the concentration of solTNFR2 per million cells and found that levels of solTNFR2 per million cells were lower in TNF KO, tmTNF KI and TNFR1 KO than in WT mice, indicating a role for TNF and TNFR1 in the shedding of TNFR2 (Figure 1c). 

### 2.2. Transmembrane TNF Controls the Expression of Membrane Bound TNFR2 in Pleural Myeloid Cells

**Figure 2:**
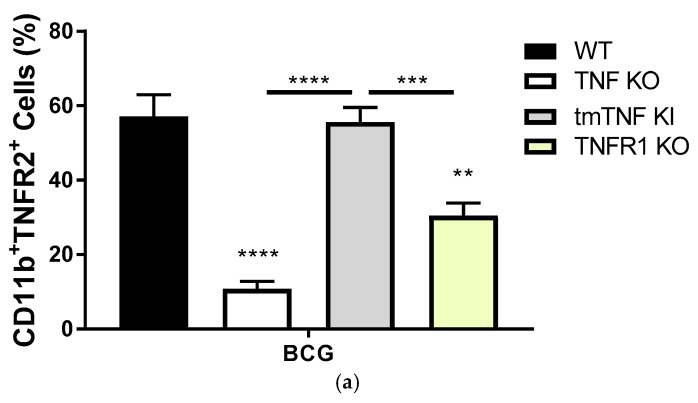

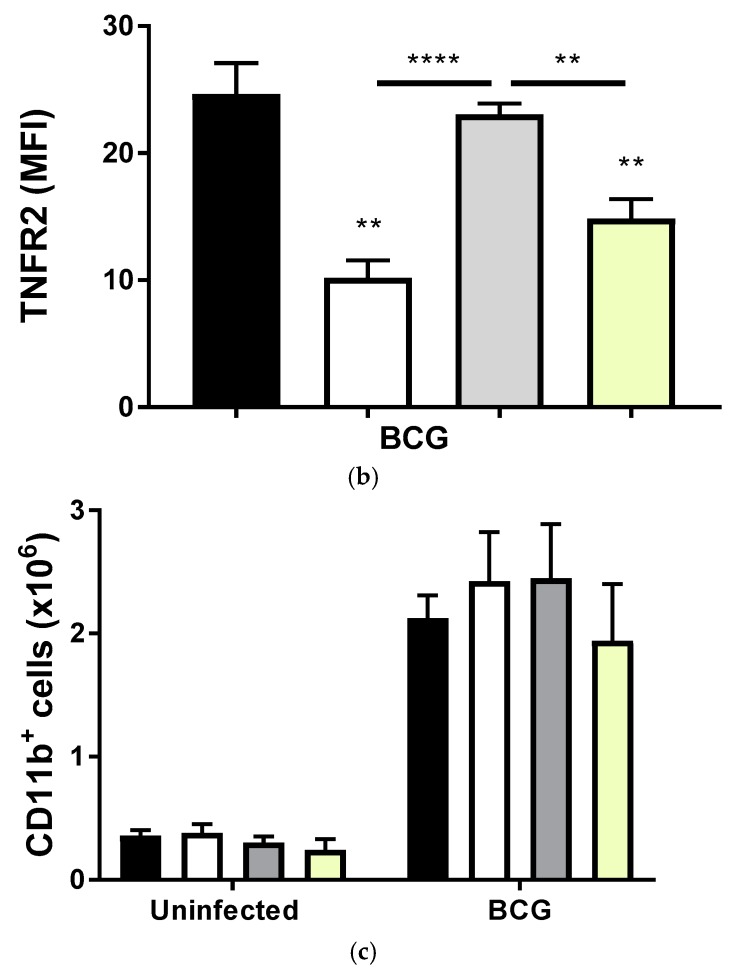
The expression of TNFR2 in pleural myeloid cells after BCG-induced pleurisy in mice. (**a**) Frequency of CD11b+ or myeloid cells expressing TNFR2; (**b**) Mean fluorescence intensity of TNFR2 expressed by pleural myeloid CD11b+ cells. Cells were isolated from the pleural cavity of BCG-infected mice at day 14 post-infection. Data are represented as bar graphs showing means +/− SEM. *n* = 9 to 11 mice/per group. Comparisons are done between infected WT mice versus infected mutant mice or as indicated in the figure. ** *p* < 0.01, *** *p* < 0.001, and **** *p* < 0.0001 versus WT if not indicated otherwise; (**c**) Absolute number of CD11+ cells isolated from the pleural cavity of uninfected or infected mice at day 14 post-infection. Data are represented as bar graphs showing means +/− SEM (*n* = 2 to 4 uninfected condition and *n* = 9 to 12 for BCG-infected mice/per group). No statistical difference is observed between mouse genotypes.

Since the concentration of solTNFR2 shed in the pleural cavity may reflect the cellular expression of membrane-bound TNFR2, we then asked whether TNF and TNFR1 regulate the expression of TNFR2 on the cell membrane of BCG-activated pleural cells. As the majority of cells recruited to the pleural cavity at day 14 after BCG-induced pleurisy are myeloid cells [17], we first evaluated by flow cytometry the frequency of myeloid cells expressing membrane bound TNFR2 and asked whether TNFR2 expression would be dependent on TNF and TNFR1 expression. The frequency of CD11b^+^TNFR2^+^ pleural myeloid cells after BCG-induced pleurisy was abrogated in the complete absence of TNF expression, but fully restored in the presence of tmTNF (Figure 2a), similar to that of WT mice, indicating that tmTNF is critical for the expression of TNFR2 on myeloid cells. Further, the absence of TNFR1 altered the percentage of cells expressing TNFR2 compared to WT mice (Figure 2a). The analysis of MFI confirmed that the expression level of TNFR2 was reduced on TNF KO cells, but restored on tmTNF cells, similar to that of WT while TNFR1 KO cells displayed statistically significant lower expression levels of TNFR2 (Figure 2b). To exclude the possibility that the different expression of TNFR2 was a consequence of the higher number of total cells recruited in the pleural cavity of mutants compared to WT mice, we assessed the amount of CD11b^+^ cells. Our data showed that the absolute number of CD11b^+^ cells in the pleural cavity was not different and independent of mouse genotypes (Figure 2c). Thus, the expression of TNFR2 on myeloid cells after BCG-induced pleurisy depends mainly on tmTNF and less on TNFR1. 

**Figure 3:**
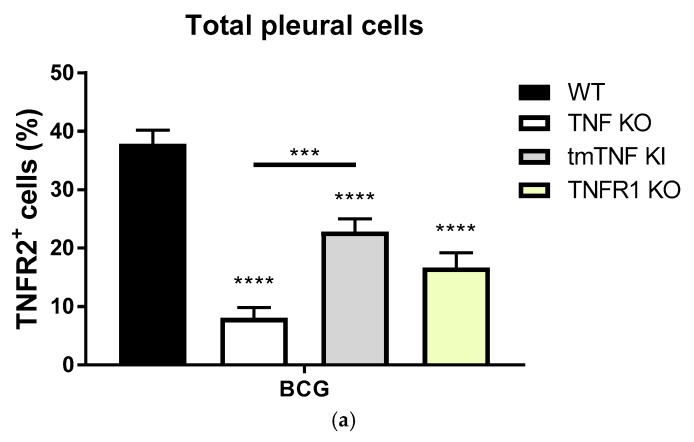

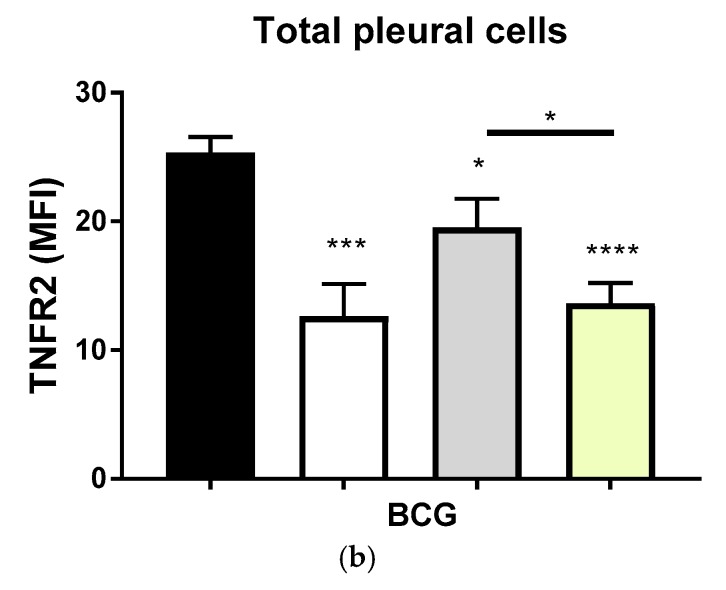
The expression of TNFR2 in the total pleural cells from BCG-induced pleurisy in mice. (**a**) Frequency of pleural cells expressing membrane TNFR2. Pleural cells from BCG-infected mice were analyzed at day 14 post-infection; (**b**) Mean fluorescence intensity of TNFR2 of pleural cells from BCG-infected mice at day 14 post-infection. Data are represented as bar graphs of means +/− SEM (*n* = 9 to 12 mice/per group). Comparisons are done between infected WT versus infected mutant mice or as indicated in the figure, * *p* < 0.05, *** *p* < 0.001, **** *p* < 0.0001.

We then evaluated the expression of TNFR2 in the total cell population of BCG-activated pleural cells. While 40% of pleural cells expressed TNFR2 after BCG infection in WT mice, the population of TNFR2^+^ pleural cells was very low in the complete absence of TNF expression, but significantly restored in the presence of tmTNF (Figure 3a). This restoration was still lower than the levels seen in WT mice, reflecting the contribution of solTNF. Interestingly, TNFR1 KO mice had a lower frequency of TNFR2^+^ cells compared with WT mice (Figure 3a). Estimation of the mean fluorescent intensity (MFI) of TNFR2 on pleural cells provided similar results, indicating that solTNF, tmTNF and TNFR1 influenced the expression of TNFR2 on the cell membrane of pleural cells (Figure 2b). Thus, the frequency, as well as the level of cellular expression of TNFR2, was affected by solTNF, tmTNF and TNFR1 during BCG-induced pleurisy. Further, these data indicate that the levels of solTNFR2 in the pleural cavity reflect with those of the cell membrane expression of TNFR2 for the total cell population and that solTNF influences the expression of TNFR2 in cell types other than the myeloid lineage.

### 2.3. BCG-Induced Pleurisy Causes Inflammation That is Controlled by Transmembrane TNF, TNFR2 and Partially by TNFR1

**Figure 4:**
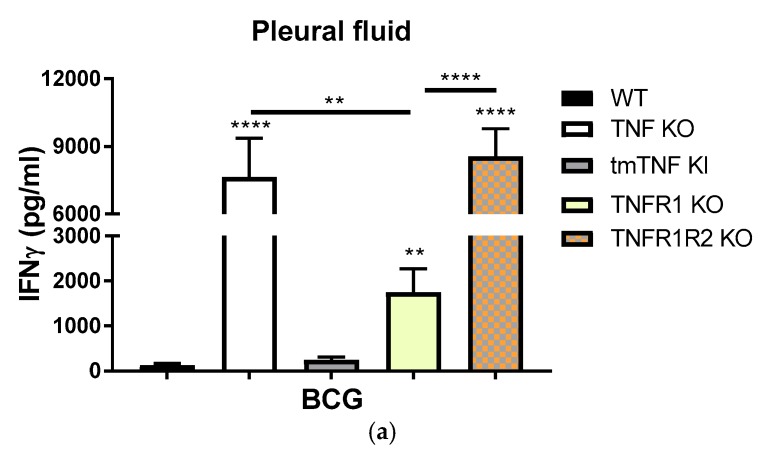

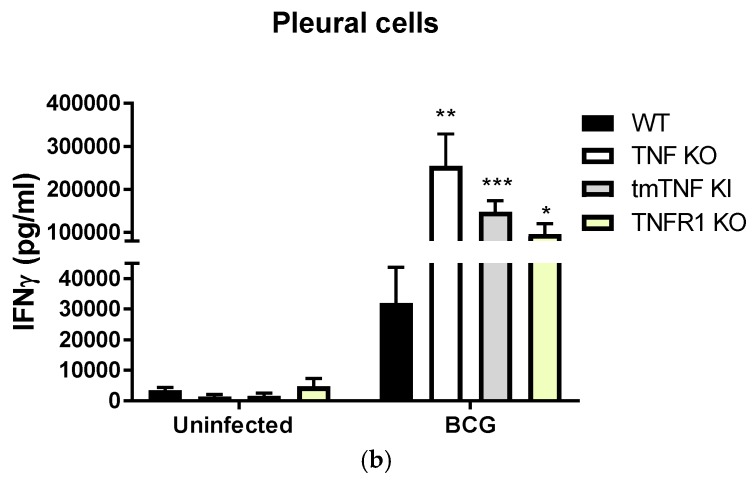
The inflammation in the pleural cavity after BCG-induced pleurisy in mice. (**a**) Concentrations of IFN-γ in the pleural cavity of BCG-infected mice at day 14 post-infection. Data are represented as bar graphs showing means +/− SEM (*n* = 12, TNFR1R2 KO *n* = 8). Comparisons are done with WT mice and as indicated in the figure ** *p* < 0.01 and **** *p* < 0.0001; (**b**) Amounts of IFN-γ measured in the supernatants of pleural cells isolated from BCG-infected mice and cultured without or with BCG (MOI 1) for 24 h. Data are represented as bar graphs showing means +/− SEM (*n* = 6–10 per group). Comparisons are done with WT mice, * *p* < 0.05, ** *p* < 0.01, *** *p* < 0.001.

We further evaluated BCG-induced inflammation in the pleural cavity of the different mouse strains by assessing IFN-γ levels. We observed exacerbated IFN-γ levels in the pleural fluid of both TNF KO and TNFR1TNFR2 KO mice (58- and 68-fold, respectively,) as compared with WT mice (Figure 4a). Mice expressing only tmTNF in the absence of solTNF were able to control pleural cavity inflammation with levels of IFN-γ similar to those of WT mice. Interestingly, even if TNFR1 KO mice displayed amounts of IFN-γ 13-fold higher than WT, this was significantly lower than TNF KO and TNFR1R2 KO mice (Figure 4a), indicating a role for both TNFR1 and TNFR2 in BCG induce pleural inflammation. These data suggest that tmTNF plays a central role in the control of the inflammation after BCG-induced pleurisy, with the contribution from both TNFR1 and TNFR2.

We then asked if the reason of the absence of IFN-γ by tmTNF was due to the incapacity of tmTNF pleural cells to produce IFN-γ rather than the ability to control the inflammation process in the pleural cavity at the time point of the infection. To answer this question, we obtained pleural cells from BCG-infected mice and cultured ex-vivo with living BCG for 24 h. Our data showed that pleural cells from tmTNF KI and TNFR1 KO produced higher levels of IFN-γ compared to WT cells, even if they produced less IFN-γ than TNF KO cells, they efficiently produced this pro-inflammatory cytokine upon reactivation (Figure 4b). These data, together, show that tmTNF KI cells are able to produce IFN-γ ex vivo but that in vivo tmTNF is crucial to control exacerbated IFN-γ, leading to the resolution of the pleural cavity inflammation which is associated with mycobacterial pleurisy.

## 3. Discussion

TNF is a pleiotropic cytokine involved in many different biological systems and in the pathogenesis of several diseases. TNF is required for host defense mechanisms against mycobacterial infections and the therapeutic blockade of TNF increases the risk of infection and reactivation of a latent infection. BCG induces the release of TNF as well as TNFRs which are also shed from the cell membrane by TACE. TACE can be activated by different stimuli including mycobacterial infections and TNF [19,20,21,22]. In vivo, TNF is only detected upon an inflammatory stimulus while soluble TNFR1 and TNFR2 are always found in the body fluids at the levels of nanograms. Moreover, activated TNF remains at the level of picograms while activated TNFR2 can reach micrograms in the serum of infected individuals. This suggests a fine tuning between TNF and its receptors which is not totally elucidated.

A majority of reported studies have explored the role of TNF and TNFRs in host defense mechanisms and the pro-inflammatory activities mediated by this potent system. However, the contribution of the TNF pathway during the resolution of a mycobacterial pleurisy is less known and merits exploration in animal models. The present study evaluates the contribution of both tmTNF and solTNF, as well as their receptors in the inflammatory process associated with BCG-induced pleurisy and its exhaustion. We explore how TNF pathway modifies the expression of both the membrane and soluble form of TNFR2 during BCG-induced pleurisy and disease resolution.

Our study shows that the recruitment of inflammatory cells caused by pleural BCG infection is associated with an important shedding of solTNFR2 which is abrogated in the absence of TNF. The pleural levels of solTNFR2 are found to be regulated by both tmTNF and solTNF as well as TNFR1. The shedding of solTNFRs in both the physiological and pathological conditions has been considered a mechanism to reduce the sensitivity of cells to TNF mediated activities by blocking TNF bioactivity [3,20,23]. Studies related to the role of TNFR2 shedding during mycobacterial infection indicate that solTNFR2 neutralizes TNF bioactivity and may impair host responses to mycobacteria as was observed in vitro and during experimental chronic infection [10,24]. Our data show that the shedding of TNFR2 is modulated by solTNF and TNFR1 during pleural infection. Interestingly, we observe that tmTNF does not affect the shedding of TNFR2 as compared with the total absence of TNF. 

Our results show that the majority of pleural myeloid cells express TNFR2 on the cell membrane which is sustained by tmTNF but surprisingly is independent of solTNF. We also observe that while tmTNF KI cells recover similar expression of membrane bound TNFR2 cells than WT cells, the absence of TNFR1 affects both the frequency of the cells expressing TNFR2 and the level of molecules expressed by myeloid cells. These data indicate that TNFR1 plays a role in the regulation of TNFR2 expression and shedding by pleural myeloid cells after BCG-induced pleurisy. Our results are in agreement with previous data reporting that the shedding of TNFR2 was mediated by TNF signaling through TNFR1 [25]. However, whereas tmTNF controls the expression of TNFR2 on the myeloid cell lineage, sol TNF influences TNFR2 expression in other cell lineages because tmTNF does not restore TNFR2 expression on all pleural cells. 

**Figure 5:**
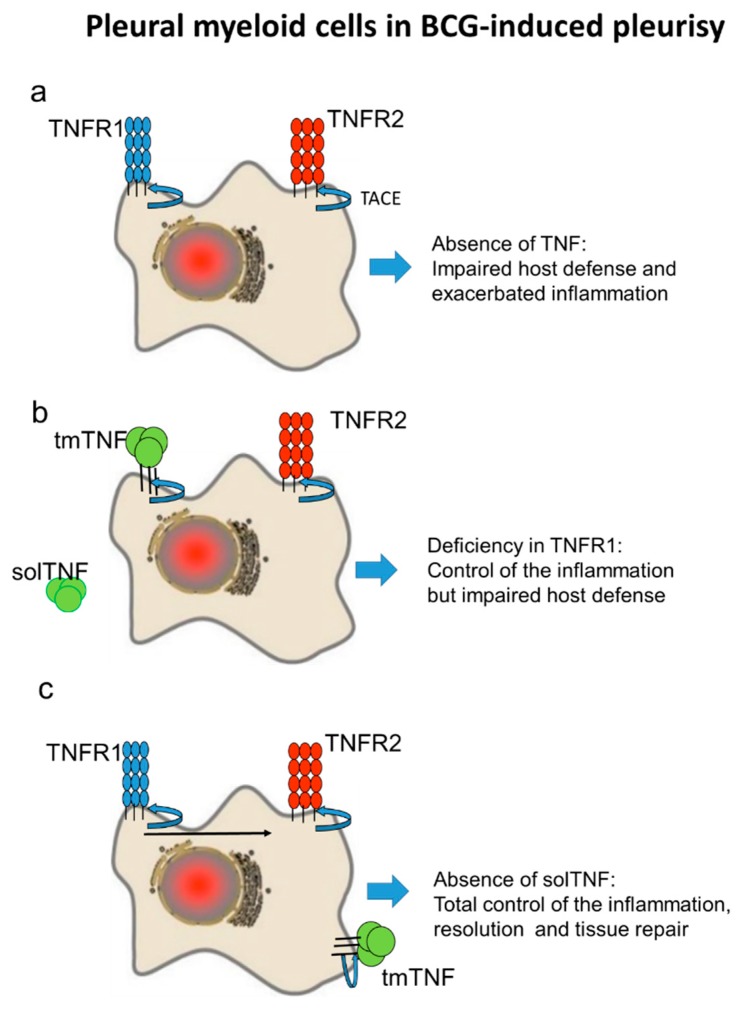
The schematic representation of myeloid cells expressing TNF and TNF receptors involved in the control of the inflammation after BCG-induced pleurisy. (**a**) The absence of TNF compromises host defense mechanisms and results in the exacerbated inflammation in the pleural cavity (**b**) TNF and TNFR2 control the pleural cavity inflammation but the absence of TNFR1 may compromise host immunity. (**c**) In the absence of solTNF, tmTNF controls the inflammation and immunity. TACE is represented as the membrane metalloprotease cleaving TNF and TNF receptors.

TNFR1 has been shown to be required for host defense against mycobacterial while TNFR2 plays a tolerogenic role during infection as it is expressed by MDSC, regulatory T cells (Tregs) and T cells [18,26,27]. However, how TNF pathway regulates inflammation and anti-inflammation in the pleural cavity is not well defined. We show that total absence of TNF leads to an exacerbated inflammation as observed during pulmonary *M. tuberculosis* infection [28]. In addition, we find that tmTNF and not soluble TNF regulates the inflammation process in the pleural cavity favoring resolution of the pleural disease (Figure 5). We also find that TNFR1 plays a role that is limited, compared to the complete absence of TNFRs leading to an exacerbation of the pleurisy and death as reported [17]. This means that TNFR2 can be the main receptor controlling the exacerbated pleurisy and suggests that tmTNF and TNFR2 control the inflammatory process although TNFR1 also plays a role in regulating the expression level of TNFR2 on pleural cells. 

We have previously reported that non-selective inhibition of both solTNF and tmTNF increases the sensibility of mice to tuberculosis infection, in contrast, selective inhibition of solTNF sparing tmTNF activities, by using dominant-negative TNF molecules, was a good strategy to maintain immunity after mycobacterial infections [29]. We and another group have also shown that treatment with anti-TNF during chemotherapy enhances mycobacterial clearance and reduces lung pathology during acute and chronic tuberculosis infection [30,31]. This suggests that the treatment with selective TNF inhibitors neutralizing only solTNF but keeping the activity of tmTNF would be a better treatment than blocking the total TNF and can be particularly indicated in cases of pleurisy where tmTNF controls inflammation.

## 4. Materials and Methods

### 4.1. Animals

Wild-type (WT) of C57BL/6 background, TNFR1TNFR2 KO and TNFR1 KO mice (The Jackson laboratory), mice deficient for TNF (TNF KO) [32], and transmembrane form TNF knockin (KI) mice (tmTNF ^Δ1–9,K11E^, deletion of amino acids 1 to 9 and substitution at position 11 or tmTNF KI) [33] were used. Adult mice (8–12 week old) housed in animal facility of the Medical Faculty, University of Geneva (Geneva, Switzerland) were used for these studies. Animal experiments were performed in agreement with institutional guidelines and were approved by the academic ethical committee on animal experimentation and the cantonal veterinary office from Geneva (authorizations N° GE/167/17 and GE/63/18, 14 December 2014 and 16 April 2018).

### 4.2. M. bovis BCG and Infection

*Mycobacterium bovis* BCG Pasteur strain 1173 P2 grown on Middlebrook 7H9 broth containing ADC (Difco) and at middle-log phase was frozen in aliquots and kept frozen at −80 °C until use. BCG-induced pleurisy was performed by intrapleural cavity injection of 10^6^ CFU of *M. bovis.* BCG in 100 µL of saline as previously reported [17]. For analyses, mice were sacrificed at day 14 post-infection and uninfected or naïve mice were also sacrificed in the same way as infected animals.

### 4.3. Pleural Cell and Fluid Preparation

The thoracic cavities were washed three times with 1 mL of 2 mmol/L EDTA-phosphate-buffered saline (PBS). After centrifugation, the first washing solution was frozen at −20 °C and used for cytokine evaluation and the pellet containing the cells were pooled to recover pleural cells [17].

### 4.4. Multiparametric Flow Cytometry Analysis

The frequency of immunological cellular subpopulations in pleural cells was analyzed by flow cytometry. Briefly, cells were stained for 30 min at 4 °C with following fluorochrome-conjugated mAb: CD11b (Clone M1/70) and TNFR2 (Clone TR75-89) (BioLegend, San Diego, CA, USA). Cells were incubated with antibodies and then washed with PBA (phosphate buffered saline containing 0.1% Sodium Azide and 1% Albumin bovine). We used a FACs CyAn flow cytometer (Beckman Coulter, Inc., Brea, CA, USA) and analyzed the data with the FlowJo (Tree Star, Ashland, OR, USA) software. We acquired 100,000 events per sample.

### 4.5. Cytokine Evaluation Enzyme-Linked Immunosorbent Assay (ELISA)

Cytokine levels in the pleural fluid and cell supernatants were assessed by Enzyme-linked immunosorbent assay (ELISA). Conditions for ELISA were optimized using anti-TNFR2 coating (purified anti-mouse CD120b, ref. 113302) and detection antibody (biotin anti-mouse CD11b, ref. 113203) from BioLegend. IFN-γ was quantified in the pleural fluid and in cellular supernatants in accordance with the manufacturer’s instructions (BioLegend). 

### 4.6. TNF Ex Vivo Stimulation of Pleural Cells

Total cells from the pleural cavity were prepared as described above. Cells were stimulated or not with BCG (multiplicity of infection MOI 1). After 24 h of culture, supernatants were recovered to quantify cytokines by ELISA.

### 4.7. Statistical Analysis

Quantitative and statistical analyses were performed using with Prism 5.0 (GraphPad Software Inc, La Jolla, CA, USA). Experiments were analyzed with an unpaired Student *t* test and multiple *t* test and corrections for multiple comparisons using the Holm-Sidak method. *p* value < 0.05 was considered as statistically significant. 

## Abbreviations

TNF	Tumor Necrosis Factor
TNFR	Tumor necrosis Factor Receptor
tmTNF	Transmembrane TNF
solTNF	Soluble TNF
solTNFR	Soluble TNFR
BCG	Bacillus Calmette-Guérin
TACE	TNF-α-converting enzyme
